# From concept to practice: intra-articular photobiomodulation for knee osteoarthritis

**DOI:** 10.3389/fimmu.2026.1793440

**Published:** 2026-03-23

**Authors:** Hao Chen, Feifei Gao, Yu Luo, Rui Wang, Arum Dwijayanti Niken, Zhifang Xu, Yi Ding, Yi Guo

**Affiliations:** 1 Research Center of Experimental Acupuncture Science, Tianjin University of Traditional Chinese Medicine, Tianjin, China; 2 School of Acupuncture & Moxibustion and Tuina, Tianjin University of Traditional Chinese Medicine, Tianjin, China; 3 National Clinical Research Center for Chinese Medicine Acupuncture and Moxibustion, Tianjin, China; 4 Tianjin Key Laboratory of Modern Chinese Medicine Theory of Innovation and Application, Tianjin University of Traditional Chinese Medicine, Tianjin, China; 5 Institute of Photoelectronic Thin Film Devices and Technology, Nankai University, Tianjin, China

**Keywords:** anti-inflammation, optical fibre acupuncture, intra-articular therapy, knee osteoarthritis, photobiomodulation

## Abstract

Knee osteoarthritis (KOA) is a prevalent degenerative joint disease worldwide. Current treatments primarily offer only symptomatic relief and lack disease-modifying efficacy. Photobiomodulation therapy (PBMT) has shown potential in modulating cellular metabolism and inflammation to alleviate KOA symptoms; however, its conventional transcutaneous delivery is significantly limited by inadequate tissue penetration and energy attenuation, leading to inconsistent clinical outcomes. To overcome these limitations, this review proposes an innovative therapeutic paradigm: intra-articular PBM. This approach involves the minimally invasive placement of miniaturized light sources—such as fibre-integrated acupuncture needles or micro-LEDs—directly into the joint cavity, enabling precise, *in situ* energy delivery to pathological tissues. Light in the 600–1000 nm range is absorbed by mitochondrial cytochrome c oxidase, initiating a cascade of biological effects including: activation of the Nrf2 pathway to enhance antioxidant capacity; suppression of the NF-κB pathway to reduce inflammation; promotion of analgesia via peripheral and central mechanisms; induction of autophagy; and inhibition of apoptosis. Together, these mechanisms contribute to chondroprotection and improvement of subchondral bone structure. Both short-term interventional and long-term implantable strategies are discussed, with emerging preclinical and early clinical evidence supporting the feasibility and efficacy of this targeted approach. Intra-articular PBM represents a significant shift from palliative symptom management to targeted, mechanism-based tissue repair. Although challenges remain in device durability, biocompatibility, and clinical translation pathways, this strategy holds considerable promise as a potential disease-modifying therapy for KOA and may offer a novel, minimally invasive treatment option for patients in the future.

## 1 Introduction

Knee osteoarthritis (KOA) is a total joint disease characterised by progressive degeneration of articular cartilage, subchondral bone sclerosis, osteophyte formation, and synovial inflammation. It represents the primary cause of pain, functional impairment, and long-term disability among middle-aged and elderly individuals globally (1). Recent analyses indicate that over 10% of the global population aged 60 and above suffer from KOA, with its rising prevalence imposing a substantial socioeconomic burden on public health systems (2). Current clinical management strategies for KOA follow a stepwise approach, encompassing early foundational interventions (e.g., weight management, physiotherapy), intermediate pharmacological treatments (e.g., non-steroidal anti-inflammatory drugs [NSAIDs]), intra-articular injections (e.g., corticosteroids, hyaluronic acid), and end-stage surgical interventions (e.g., joint replacement) (3). However, these mainstream therapies still have some limitations. Long-term NSAID use frequently induces gastrointestinal, cardiovascular, and renal adverse reactions; intra-articular injections provide short-term symptom relief but cannot reverse cartilage damage, whilst repeated injections carry infection risks that may accelerate cartilage degeneration; total knee arthroplasty represents a highly invasive, costly last resort associated with infection, thrombosis, and prosthesis loosening risks (4). More fundamentally, these therapies represent palliative symptomatic management lacking disease-modifying effects. Despite substantial global research investment, the clinical development failure rate for true disease-modifying osteoarthritis drugs (DMOADs) remains exceedingly high, with over 90% of candidate drugs failing to achieve clinical translation (5, 6). This underscores the complexity of KOA’s pathological mechanisms and the urgent need to develop novel therapeutic paradigms.

Photobiomodulation therapy (PBMT), also known as low-level laser therapy (LLLT), has demonstrated unique potential as a non-pharmacological, extracorporeal photobiomodulation (PBM) treatment modality in the management of KOA (7). In recent years, multiple systematic reviews and meta-analyses have provided support for the clinical application of PBMT (8, 9). For instance, a recent network meta-analysis incorporating 32 randomised controlled trials (RCTs) evaluated the efficacy of various physical therapies for KOA, including electrical stimulation therapy, LLLT, thermotherapy, cryotherapy, and extracorporeal shock wave therapy, with resistance and range-of-motion exercises serving as the control group. The results indicated that LLLT effectively reduced visual analogue scale (VAS) pain scores and improved Western Ontario and McMaster Universities Osteoarthritis Index (WOMAC) functional scores in KOA patients, affirming its value in KOA management (10).

Nevertheless, the clinical application of PBMT in KOA remains controversial, with its heterogeneous efficacy drawing considerable attention. A critical technical bottleneck lies in the limited tissue penetration depth of light. During transcutaneous irradiation, light energy undergoes significant scattering and attenuation as it traverses multiple tissue layers—including skin, subcutaneous fat, and the joint capsule—resulting in effective energy densities reaching deep pathological areas (such as cartilage and synovium) that may fall far below the threshold required to induce biological stimulation effects (11). This issue of inadequate energy delivery, coupled with the lack of standardisation in treatment parameters (such as wavelength, power, and energy density), and variations in individual anatomical structures and tissue optical properties, collectively contributes to inconsistent clinical research outcomes, limiting the maximisation of PBMT efficacy (8). Notably, an observational clinical study suggests that combined intra-articular red and infrared laser irradiation under ultrasound guidance yields favourable repair outcomes, providing preliminary clinical rationale for the concept of “intra-articular PBM” (12).

Against this backdrop, intra-articular PBM has emerged as a novel paradigm designed to overcome the limitations of conventional transcutaneous irradiation. However, a systematic review of this emerging field remains lacking, one that elucidates its scientific basis, technical pathways, preclinical evidence, and clarifies its translational prospects. Consequently, this paper aims to comprehensively address the innovative concept of “intra-articular PBM for KOA” for the first time. By synthesising multidisciplinary knowledge, it elucidates the scientific rationale and translational feasibility of intra-articular PBM as a potential disease-modifying therapy for KOA. This provides researchers, clinicians, and industry stakeholders with a clear roadmap to accelerate the translation of this novel therapeutic strategy from bench to bedside, ultimately offering new hope for patients with KOA.

## 2 Scientific basis for PBM in KOA treatment

The efficacy of PBMT rests upon well-established physical and molecular foundations governing light-biological tissue interactions. The following sections will delve into the scientific principles underpinning PBM for KOA treatment, systematically elucidating its potential disease-modifying mechanisms—from light propagation within tissues to the cellular and molecular cascades it initiates.

### 2.1 Interaction between light and tissue/cells

The therapeutic efficacy of PBM primarily depends on the effective delivery of light energy to target tissues. When light of specific wavelengths irradiates biological tissues, key physical processes such as absorption, scattering, and transmission occur (13). Haemoglobin and melanin predominantly absorb blue and green light, exhibiting weaker absorption of red or near-infrared light (approximately 600–1000 nm). This enables red and near-infrared light to penetrate deeper tissues with less energy attenuation (14). However, even within the “optical therapeutic window”, light undergoes significant scattering when traversing tissues such as skin and subcutaneous fat, causing energy density to decline sharply with increasing depth (15). This represents a major physical limitation for transdermal PBM therapy targeting deep-seated joint pathologies (11).

At the cellular level, the therapeutic effects of PBM originate from the highly efficient absorption of photons at specific wavelengths by target chromophores within the cell. There is substantial evidence that light within the 600–1000 nm wavelength range is absorbed by cytochrome c oxidase (CCO), also known as Complex IV. CCO is the terminal enzyme in the mitochondrial electron transport chain (ETC) (16–18). This photon absorption induces a conformational change in CCO, causing the dissociation of its inhibitory ligand, nitric oxide (NO) (19). This pivotal event has a dual effect: it reverses NO’s suppression of cellular respiration while simultaneously releasing NO itself. NO then functions as a regulatory signaling molecule (20). Activation of CCO ultimately enhances ETC efficiency, strengthens mitochondrial membrane potential, and promotes ATP synthesis, providing cells with a robust energy foundation (21).

**Figure 1:**
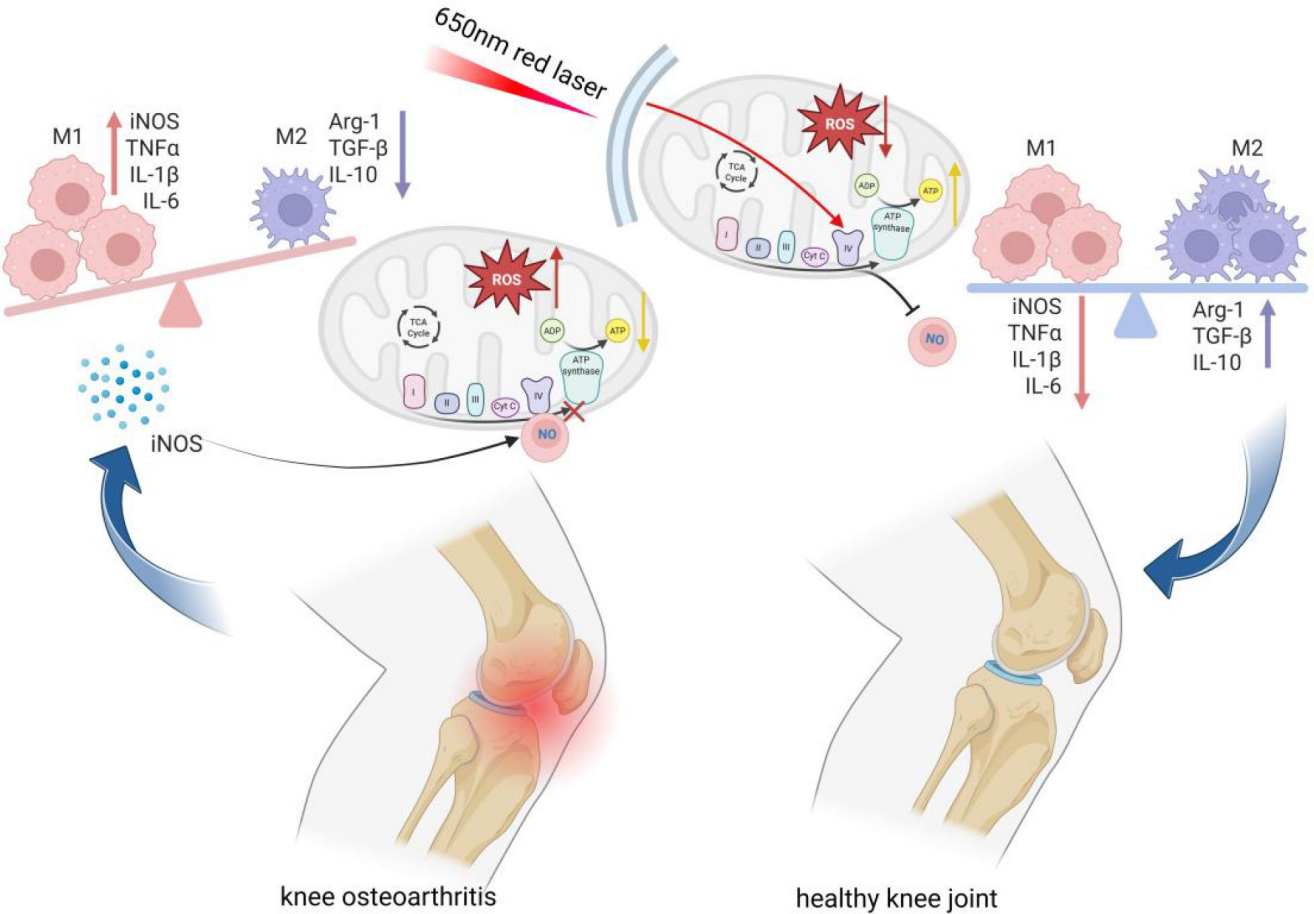
Mechanism diagram of intra-articular photobiomodulation.

**Figure 2:**
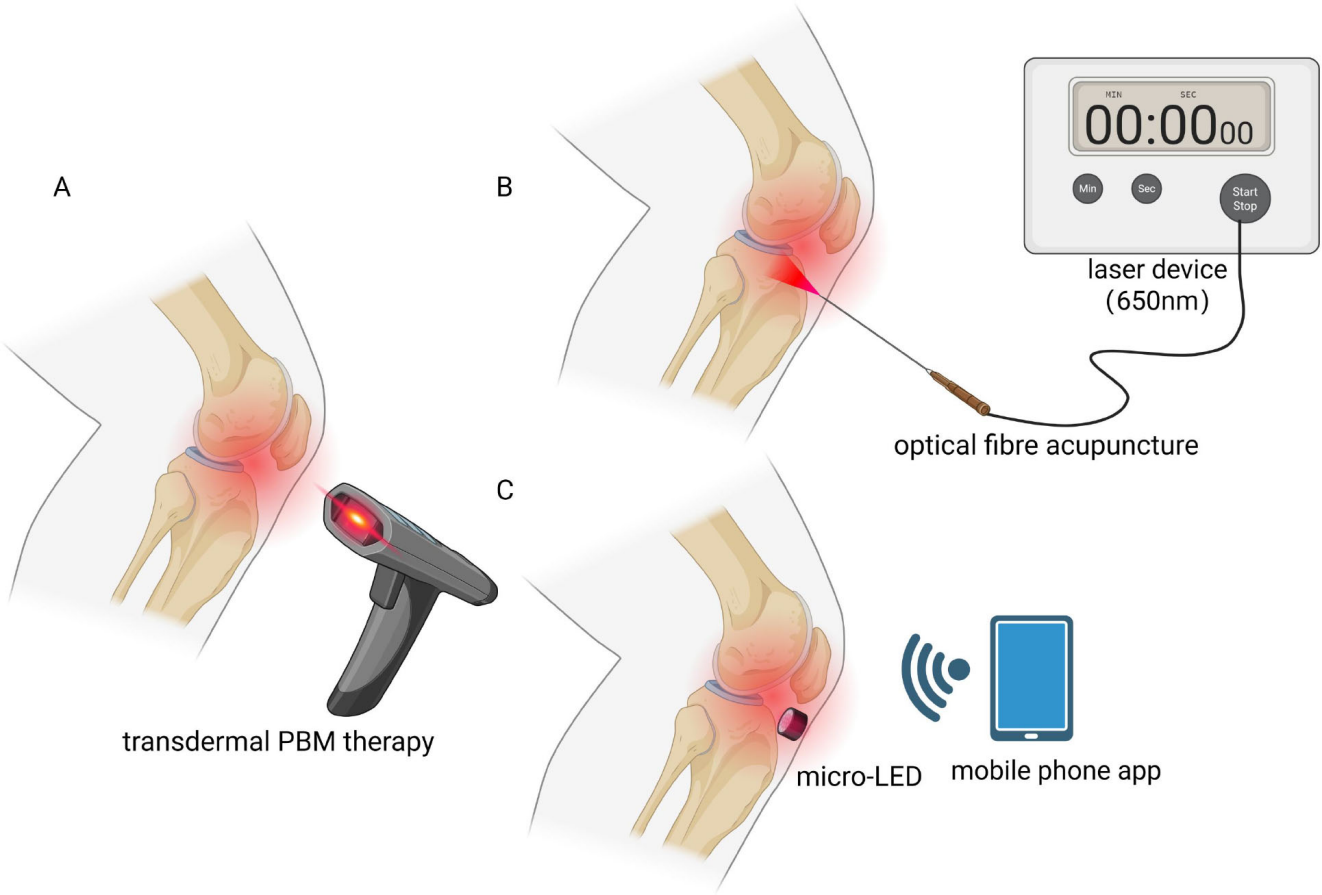
Design concept of intra-articular photobiomodulation. **(A)** shows conventional transdermal photobiomodulation therapy. **(B, C)** illustrate the schematic designs of the intra-articular photobiomodulation using optical fibre acupuncture and micro-LEDs, respectively. PBM, photobiomodulation.

During PBM for KOA, this molecular cascade activates CCO in synovial macrophages, releasing NO that collectively restarts mitochondrial respiration and initiates downstream signalling networks (22). Concurrently, enhanced ETC function converts pathologically accumulated reactive oxygen species (ROS) into physiological signalling molecules, thereby restoring redox homeostasis. The resulting enhancement in cellular energy metabolism (manifested as increased ATP production), synergising with NO-mediated and other signalling events, ultimately drives the phenotypic shift of macrophages from pro-inflammatory M1 to reparative M2 states (23). This integrated mechanism provides a systematic molecular biological explanation for how optical fibre acupuncture (OFA)-mediated intra-articular PBM alleviates synovial inflammation (**Figures 1**, **2**).

### 2.2 Molecular mechanisms of PBMT for KOA

**Figure 3:**
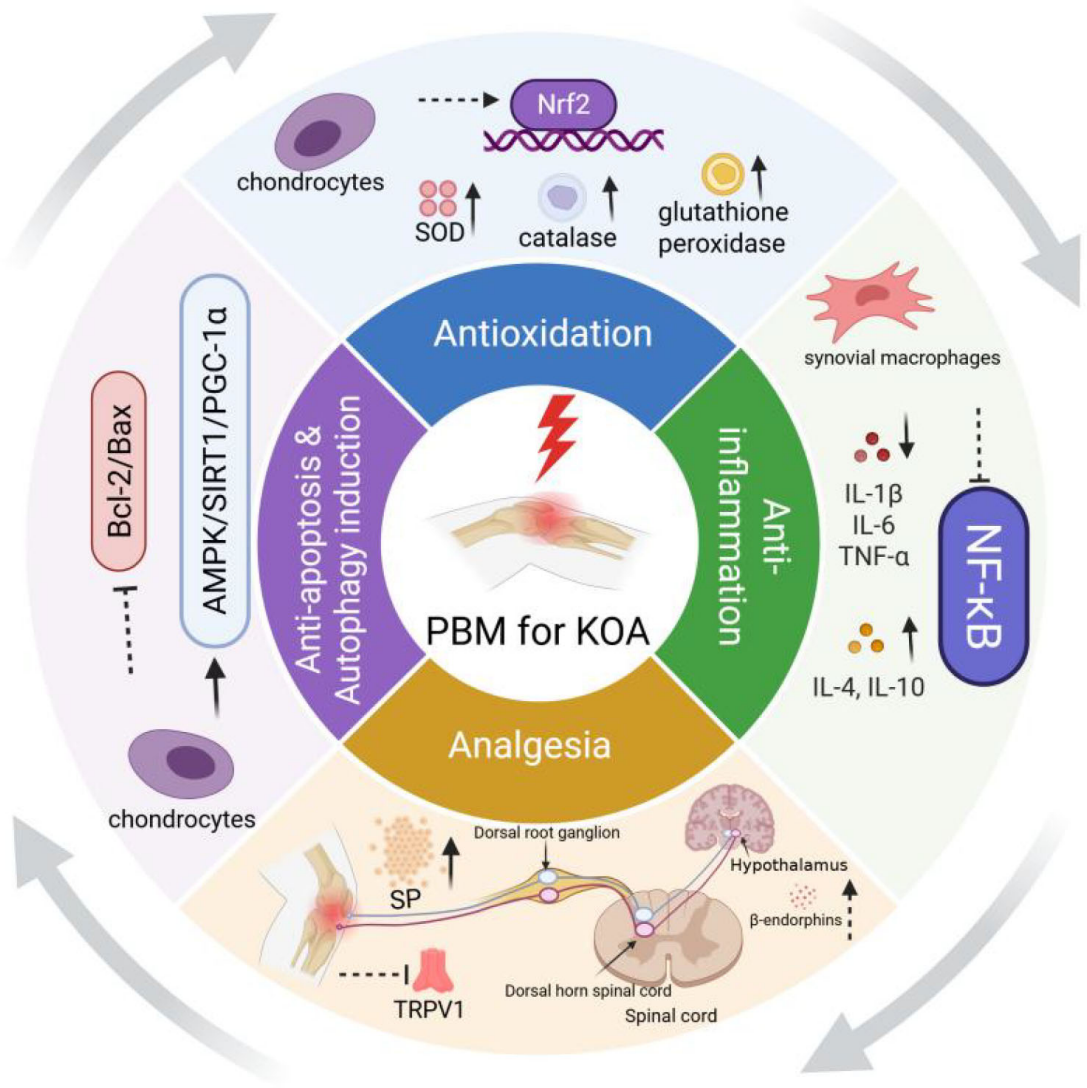
Potential mechanism of photobiomodulation for knee osteoarthritis. PBM, photobiomodulation; KOA, knee osteoarthritis; IL, interleukin; TNF, tumor necrosis factor; NF-κB, nuclear factor kappa-B; Nrf2, nuclear factor erythroid 2-related factor 2; SOD, superoxide dismutase; TRPV, transient receptor potential vanilloid; SP, substance P; Bcl-2, B-cell leukemia/lymphoma 2; Bax, Bcl2-associated X; AMPK, adenosine 5′-monophosphate (AMP)-activated protein kinase; SIRT, silent mating type information regulation 2 homolog; PGC, Peroxisome proliferator-activated receptor gamma coactivator.

PBM initiates a cascade of signal transduction events by activating CCO, intervening in the pathological progression of KOA through multiple key pathways (**Figure 3**).

#### 2.2.1 Antioxidant effects

Oxidative stress constitutes a pivotal component in the pathogenesis of KOA. A review by de Freitas et al. systematically elucidated this mechanism, noting that although PBM transiently and mildly increases ROS, this serves as an excitatory stimulus signal. It activates key transcription factors such as Nrf2, thereby significantly enhancing the activity of endogenous antioxidant enzymes including superoxide dismutase (SOD), catalase, and glutathione peroxidase (24). Direct evidence was provided by Yamada et al.’s animal experiments (25). In a KOA rat model, a 904 nm wavelength laser was applied directly at a perpendicular angle to the medial and lateral skin over the joint, administered three times weekly for a total of eight treatments. Results demonstrated that PBM (particularly at the 18 J/cm² dose) markedly reduced levels of the lipid peroxidation product, malondialdehyde (MDA) and protein carbonyls, significantly increased SOD activity, particularly in the spinal cord and brainstem, and modulated the content of non-protein thiols such as glutathione. Concurrently, it reduced myeloperoxidase (MPO) activity in joint lavage fluid, suggesting its capacity to inhibit neutrophil infiltration and activation, thereby mitigating local oxidative damage. Furthermore, it suppressed nitric oxide synthase (NOS) activity, diminishing the toxic effects of NO and enhancing overall antioxidant capacity. Martins et al. (26) employed 630 nm LED irradiation on the knee joints of KOA model rats, observing that this treatment significantly increased the activity of endogenous antioxidant enzymes (SOD and catalase) in OA joints, reduced levels of lipid peroxidation damage markers such as thiobarbituric acid reactive substances (TBARS), and effectively mitigated oxidative stress-induced damage to chondrocytes.

#### 2.2.2 Anti-inflammatory and analgesic effects

One characteristic of osteoarthritis is persistent low-grade inflammation within the synovium and cartilage. Extensive *in vivo* and *in vitro* studies confirm that PBM exerts significant anti-inflammatory effects by modulating key inflammatory pathways, with its core mechanism centred on inhibiting the activation of the nuclear factor κB (NF-κB) signalling pathway. For instance, research by Sakata et al. (27) demonstrated that high-frequency near-infrared laser irradiation at an energy density of 8 J/cm² on interleukin (IL)-1β-stimulated human primary chondrocytes specifically inhibits the NF-κB signalling pathway activated by IL-1β. This manifests as reduced phosphorylation, prevented nuclear translocation, and diminished DNA-binding activity, thereby significantly downregulating the expression of key inflammatory factors such as IL-1β, IL-6, and tumor necrosis factor (TNF)-α at the protein expression level. Concurrently, PBM positively modulates anti-inflammatory responses. Tim et al. (28) observed in a rat KOA model that laser irradiation (808 nm, 1.4 J) of the knee joint markedly reduced expression of the key pro-inflammatory factor IL-1β whilst significantly upregulating anti-inflammatory factors IL-4 and IL-10. This bidirectional modulation of pro-inflammatory and anti-inflammatory balance effectively remodelled the intra-articular inflammatory microenvironment, creating favourable conditions for subsequent tissue repair.

Concurrently, Fan et al. (29) employed a weight-bearing imbalance test to evaluate PBM’s analgesic effects. Results indicated that mice in the PBM group (810 nm, 39 J/cm²) exhibited markedly improved pain-related behaviour from the fourth week post-treatment, manifested as reduced weight-bearing asymmetry between the affected and unaffected limbs. Furthermore, *in vitro* and *in vivo* experiments demonstrated that PBM downregulated the protein expression levels of IL-1β and IL-6 within synovial tissue. These cytokines are key mediators of pain and inflammation in osteoarthritis, and the analgesic effect is closely associated with reduced levels of inflammatory mediators (IL-1β and IL-6). By reducing local concentrations of pain-inducing substances such as substance P through the aforementioned anti-inflammatory mechanisms, PBM effectively diminishes peripheral sensitisation of pain receptors. More directly, PBM can influence specific ion channels. Chow et al. (30) confirmed that 830 nm laser irradiation reduces the activity of transient receptor potential vanilloid type 1 (TRPV1) in dorsal root ganglion neurons of rats and inhibits fast axonal transport, providing electrophysiological evidence for PBM’s direct intervention in pain signal transmission. At the central level, PBM can stimulate the endogenous analgesic system. Hagiwara et al. demonstrated that 830 nm laser irradiation of rat paws upregulates gene expression of the β-endorphin precursor (proopiomelanocortin, POMC) and its regulatory factor (corticotropin-releasing factor, CRF), significantly elevates plasma β-endorphin levels, and enhances the pain threshold to thermal stimulation. This effect was blocked by the opioid receptor antagonist naloxone, confirming that PBM produces central analgesia by stimulating the release of endogenous opioids (31).

#### 2.2.3 Anti-apoptotic and autophagy-promoting effects

PBM counteracts chondrocyte loss and functional decline in KOA by maintaining cellular homeostasis. Its anti-apoptotic action centres on the mitochondrial pathway. For example, research on skeletal muscle satellite cells demonstrated that He-Ne laser (632.8 nm) upregulates the anti-apoptotic protein Bcl-2 and downregulates the pro-apoptotic protein Bax, thereby stabilizing mitochondrial membrane potential, reducing cytochrome c release, and ultimately inhibiting caspase-3 activation (32, 33). Since the Bcl-2/Bax balance is a core conserved mechanism regulating mitochondrial apoptosis, PBM may exert anti-apoptotic effects in chondrocytes through similar molecular pathways, thereby reducing programmed cell death in the pathological environment of KOA. Moreover, PBM serves as an effective inducer of cellular autophagy. Under KOA pathological conditions, impaired autophagic flux leads to abnormal accumulation of proteins and organelles. Xue et al. demonstrated that a near-infrared laser-responsive nanomedicine delivery system, when combined with light irradiation (808 nm, 1 W/cm², 600 s), significantly activates the AMPK/SIRT1/PGC-1α signalling pathway in chondrocytes. This upregulates the expression of the autophagy marker LC3-II and reduces p62 accumulation, thereby enhancing the autophagy flux (34). This process facilitates the clearance of damaged mitochondria and misfolded proteins by chondrocytes, thereby maintaining intracellular homeostasis and delaying cellular senescence and degenerative changes.

## 3 Concept and practice for intra-articular PBMT of KOA

### 3.1 Fundamental solutions for intra-articular energy delivery

In recent years, the modification of acupuncture needles into multifunctional, intelligent drug and energy delivery vehicles has emerged as a rapidly developing research direction in KOA treatment. Early innovations focused on point-to-point drug delivery, such as nanodrug-delivery acupuncture (nd-Acu) or microneedle delivery technologies. By loading anti-inflammatory and tissue-repair molecules onto the acupuncture, these approaches achieved synergistic therapy where “the needle delivers the drug upon insertion”, establishing a new paradigm for localised, precise drug administration in KOA (35, 36). This successful approach inspired researchers to further expand its functional boundaries: if the acupuncture can deliver drugs, could it also serve as a conduit for precise energy delivery (such as light energy)? This led to the conception of the OFA. By integrating micro-optical fibres within the acupuncture needle, it innovatively combines the minimally invasive intervention capability of acupuncture with the optical therapeutic potential of PBM, achieving “light follows the acupuncture”. This functional leap from “drug delivery” to “light delivery” fundamentally resolves the core challenge of traditional transcutaneous PBM—limited efficacy due to tissue attenuation—and provides an ideal technical vehicle for intra-articular PBM.

The cited studies collectively demonstrate the technical feasibility and precision-targeting capability of OFA as a platform for deep-tissue energy delivery, which forms a foundational rationale for its application in intra-articular PBM for KOA. In fundamental studies, the Shanghai University team utilised the OFA placed in the tail vein of rats to achieve real-time monitoring of drug concentrations *in vivo* (37). Additionally, the team stimulated the Zusanli acupoint (ST36) in mice for 10 minutes using both a 633nm OFA at 10mW compared with conventional acupuncture. Raman spectroscopy was employed to analyse changes in blood components (such as lipids, glucose, and NADH). The results indicated that both stimulation methods led to reduced concentrations of porphyrin, NADH, lipids, and glucose in the blood, while simultaneously increasing the concentration of phosphatidylinositol (38). This validates the OFA’s capacity for precise biological interfacing and controlled energy delivery. Jeon et al. (39) compared therapeutic efficacy in Parkinson’s model mice following targeted stimulation of the Yanglingquan acupoint (GB34) with OFA at different wavelengths (650/830 nm, 10 min). They concluded that the 830 nm OFA effectively ameliorated Parkinson’s symptoms by protecting dopaminergic neurons and reducing neuroinflammation in the brain. This confirms that OFA-mediated energy can effectively modulate deep-seated pathological processes. The RCTs by Kim’s team (40, 41) translate this technical capability into a clinically validated, safe, and effective intervention for a musculoskeletal condition (chronic low back pain). This demonstrates the direct clinical applicability of the OFA platform for managing pain and dysfunction originating from deep tissues. While these studies do not directly target the knee joint, they substantiate the core principles required for intra-articular therapy: 1) precise delivery of energy to a defined deep target, 2) the ability to trigger relevant biological responses (anti-inflammatory or neuromodulatory), and 3) a viable clinical safety profile. Therefore, they provide a compelling technological and mechanistic precedent.

### 3.2 Research progress on intra-articular PBM for KOA

Intra-articular PBM for KOA is an emerging field. However, recent years have yielded a series of research findings spanning *in vitro* studies, animal models, and preliminary clinical explorations (**Table 1**). While most studies remain at the preclinical stage, they collectively point to the substantial potential of this strategy in mitigating pathological progression and promoting repair in KOA.

**Table 1:** Application of optical fibre acupuncture in animals and patients.

Ref.	Animals/patients	Points	Parameters	Main findings	Results
Zhang H et al., 2016 (37)	SD rat	The tail vein of an anaesthetised rat	633 nm	Raman peak positions for levofloxacin lactate at 1404 cm^−1^, 1574 cm^−1^, and 1621 cm^−1^	The concentration of levofloxacin lactate in the blood was measured *in vivo* and in real-time.
Liu S et al., 2018 (38)	Male BALB/c mice	ST36	633 nm, 10 mW, 10 min	The intensities of Raman bands for porphyrin ↓	Reduce the concentration levels of porphyrin, NADH, lipids and glucose, whilst increasing the concentration level of phosphatidylinositol in blood.
Kim JH et al., 2022 (40)	Patients with chronic non-specific low back pain	GB30, BL23, BL24, and BL25	650 or 830 nm, 10 min	VAS, ODI, and EQ-5D-5L scores ↓	The 650 nm and 830 nm wavelengths can be employed for the treatment of chronic non-specific low back pain.
Chen JL et al., 2022 (12)	Patients with knee osteoarthritis	The knee joint	20 minutes of red (658 nm, 50 mW), 10 minutes of infrared (810 nm, 100 mW)	The Lequesne index ↓; The volume of suprapatellar SF and SF proteins associated with inflammation ↓	A viable alternative for elderly patients with knee osteoarthritis unsuitable for surgical interventions or intra-articular platelet-rich plasma injections.
Jeon H et al., 2025 (39)	Mice with MPTP-induced PD	GB34	650 or 830 nm, 10 min	830 nm: motor performance ↑; the nigrostriatal TH-positive immunoreactivities ↑; α-synuclein ↓; Bax/Bcl2 ↓; IL-1β, IL-6, and TNF-α ↓; IL-10 ↑; nigrostriatal astrocyte ↓; microglia activation ↓	The 830 nm effectively ameliorates PD symptoms by protecting dopaminergic neurons and reducing neuroinflammation in the brain.
Hong Y et al., 2025 (41)	Patients with non-specific chronic low back pain	GB30, BL23, BL24, and BL25	650 nm, 20 mW, 10 min	VAS scores (*p* = 0.0021, visit 9; *p* = 0.0015, visit 10) ↓	Provide strong clinical evidence of the safety and efficacy of 650 nm ILA for managing non-specific chronic low back pain.
Seo SH et al., 2025 (45)	Monosodium Iodoacetate induced OA rat model	GB34 and GB39	650 nm, 10 mW or 20 mW, 50 Hz, 3 min	10 mW: hind paw weight distribution ↑; bone volume fraction, trabecular volume, cortical bone area, and cortical bone thickness ↑; muscle fiber area ↑	The 10 mW laser acupuncture proves more effective than 20 mW in mitigating joint damage and preserving musculoskeletal tissue.
Kamau VN et al., 2025 (46)	Monosodium iodoacetate- induced OA rat model	GB34	830 nm, 50 Hz, 20 mW, 3 min	paw withdrawal thresholds ↑; ossicle volume and area ↓; cartilage damage scores ↓	Laser acupuncture could potentially improve pain hypersensitivity, bone structure, and cartilage degradation.

NADH, nicotinamide adenine dinucleotide; CNLBP, chronic non-specific low back pain; VAS, visual analog scale; ODI, Korean version of the Oswestry Disability Index; EQ-5D-5L, European Quality of Life Five Dimension Five Level Scale; OA, osteoarthritis; SF, synovial fluid; PD, Parkinson’s disease; ILA, invasive laser acupuncture.

#### 3.2.1 *In vitro* study

*In vitro* studies provide a direct cellular biological basis for optimizing therapeutic parameters (e.g., wavelength, energy density) for intra-articular PBM. Using human or animal-derived chondrocytes or cartilage tissue blocks under simulated intra-articular close-range irradiation conditions, studies demonstrate that PBM at specific parameters can circumvent tissue attenuation associated with transcutaneous irradiation. This enables the stimulation of more potent and specific cellular responses with lower energy input. The core mechanism lies in synergistically promoting anabolic processes while inhibiting catabolic processes, thereby jointly maintaining cartilage homeostasis. To screen optimal PBM parameters, one study employed TNF-α-stimulated rat primary synovial fibroblasts (FLS) to simulate an inflammatory environment. Irradiation was conducted at fixed irradiance (44 mW/cm²) using four wavelengths (625, 810, 940, 1050 nm) and energy densities ranging from 13 to 78 J/cm². The anti-inflammatory effects of different parameters were evaluated by quantifying mRNA expression levels of key pro-inflammatory factors (IL-1β, IL-6, COX-2, and iNOS) via RT-qPCR. Among all tested parameters, irradiation at 810 nm with 39 J/cm² demonstrated the most significant suppression of pro-inflammatory gene expression (IL-1β, IL-6, COX-2, iNOS) in FLS (28). This identifies the optimal candidate wavelength and dose for *in vivo* studies. Regarding the mechanism of PBM’s action on chondrocytes, studies confirm that PBM upregulates the gene and protein expression of type II collagen and aggregated proteoglycans, significantly promoting chondrocyte proliferation and activity (42). The key regulatory factor for this anabolic effect is the transcription factor Sox9, whose expression is also significantly enhanced following PBM (43). The upregulation of Sox9 directly drives the synthesis of cartilage-specific extracellular matrix (ECM), providing the material foundation for cartilage repair. Concurrently, PBM effectively curbs ECM degradation through its anti-inflammatory action. In models stimulated by the inflammatory cytokine IL-1β, PBM significantly suppressed the expression of key matrix-degrading enzymes (such as ADAMTS5 and MMP-13) and the pro-inflammatory factor TNF-α (44). This inhibition of catabolic processes prevents premature degradation of newly synthesised matrix. Consequently, *in vitro* experiments reveal that PBM, through close-range efficient irradiation, simultaneously activates the Sox9-centred ECM synthesis pathway while suppressing the inflammation-catabolism pathway mediated by NF-κB and others. The synergistic interaction of these pathways enables “synthesis exceeding degradation” of the ECM, ultimately achieving a net accumulation of ECM, enhanced cellular vitality, and anti-apoptotic effects at the cellular level. This provides a robust theoretical basis for intra-articular PBM promoting cartilage repair and delaying degeneration *in vivo*.

#### 3.2.2 *In vivo* study

*In vivo* studies conducted in KOA animal models constitute a critical component in evaluating the efficacy and safety of intra-articular PBM. Although not entirely equivalent to direct intra-articular irradiation, the clinical advancement of OFA technology—a highly practicable form of intra-articular PBM that delivers light precisely to deep acupoints or periarticular tissues—provides compelling evidence for this field. A comparative analysis of two pivotal studies highlights key parameters influencing therapeutic outcomes. For instance, Seo et al. (45) demonstrated that eight sessions of OFA (650 nm, 10 mW) over four weeks compared to a 20 mW regimen, targeting GB34 and Xuanzhong acupoint (GB39) for three minutes per point, following intervention, cartilage degeneration and proteoglycan content were assessed via carmine staining. Pain-related protective weight-bearing behaviour was quantified using hindlimb weight-bearing distribution testing. Micro-CT analysis evaluated bone volume fraction, trabecular bone volume, and cortical bone thickness in the distal femur or tibial plateau. Muscle atrophy was assessed through histological examination and measurement of wet weight in the gastrocnemius and quadriceps muscles. Results indicated that OFA at 10 mW significantly alleviated pain responses in KOA rats, protected cartilage and bone structures, and delayed muscle atrophy. This underscores the existence of an optimal therapeutic window rather than a simple “more energy equals better outcome” relationship. Kamau et al. (46) compared the efficacy of electroacupuncture, bee venom acupuncture, and OFA therapy (830 nm, 20 mW, 3 min, bilateral GB34 acupoint, three weekly sessions over four weeks) in KOA model rats. Mechanical hyperalgesia was assessed weekly via paw withdrawal threshold testing. Following treatment, micro-CT imaging analysed bone microarchitecture. Histological evaluation examined cartilage integrity. Results indicated significant analgesic effects from weeks 2–3 onwards for both electroacupuncture and OFA. By week 4, electroacupuncture and OFA demonstrated optimal and consistent effects in preserving joint structure and maintaining cartilage integrity, exhibiting extremely significant differences compared to the OA model group. The use of a longer wavelength (830 nm vs. 650 nm) may engage different tissue chromophores and photobiological pathways, potentially favoring effects on subchondral bone. These related studies further indicate that this technique represents a non-pharmacological therapeutic strategy with broad application prospects. The logical next step is to translate these parameter-dependent effects into optimized protocols for direct intra-articular light delivery in KOA, where precise control over energy delivery is paramount.

#### 3.2.3 Clinical study

Currently, rigorous, targeted intra-articular PBM clinical trials remain limited, though preliminary feasibility signals emerge from related clinical reports and analogous applications. A pioneering exploratory clinical study (12) documented the feasibility and preliminary efficacy of ultrasound-guided intra-articular laser irradiation in elderly patients with KOA. This technique involved inserting a flexible optical fibre guidewire through a hollow needle into the knee joint. Under musculoskeletal ultrasound guidance, the fibre was advanced along the lateral patellar tendon pathway to deliver 20 minutes of red light laser irradiation (658 nm, 50 mW) followed by 10 minutes of infrared light laser irradiation (810 nm, 100 mW) to the osteoarthritic knee joint. Treatment was administered every two weeks for a total of three sessions, with follow-up assessments conducted at 1, 3, and 6 months post-treatment. Results demonstrated significant improvements in patients’ WOMAC pain and functional scores following therapy, with the procedure exhibiting good safety profiles and no reported infections or other serious adverse events. Although limited by small sample size and lack of controls, this study represents the first human validation of the technical feasibility and clinical potential of intra-articular laser therapy. Additionally, RCTs by Kim’s team (40, 41) demonstrated significant efficacy of 650 nm OFA for chronic low back pain.

### 3.3 Interventional and implantable light sources

The miniaturisation and biocompatibility of light sources are prerequisites for achieving intra-cavity therapy. Fibre-optic light delivery systems represent an alternative and more mature technical pathway. This system avoids implanting the light source itself within the body, instead guiding light generated by an external laser to the target location within the joint cavity via extremely fine, flexible optical fibres. This approach avoids the thermal management and energy supply challenges associated with implanted sources, making it particularly suitable for short-duration, high-energy interventional therapies. The OFA—an innovation integrating optical fibres within acupuncture needles—enables precise photostimulation of acupoints or deep tissues (37–41). Additionally, micro-light-emitting diodes (Micro-LEDs) have emerged as viable options for *in-vivo* illumination systems. Their advantages include micrometre-scale dimensions, facilitating integration onto flexible substrates to conform to joint surfaces; extremely low power consumption, making them suitable for implantable applications; and selectable wavelengths that precisely match the absorption peaks of photoreceptors such as CCO (e.g., 630 nm, 810 nm, 850 nm) to achieve specific biological effects (47, 48). Researchers have developed flexible, stretchable Micro-LED patches capable of delivering large-area irradiation across human skin surfaces to promote wound healing (49, 50). In addition, investigations are underway into biodegradable or absorbable LEDs fabricated from biocompatible polymeric materials. These devices can safely degrade within the body post-treatment, eliminating the need for secondary surgical removal and significantly enhancing therapeutic safety (51, 52). Moreover, photofluidic chips integrate microfluidics with optical technology, enabling precise light control and sensor integration by guiding light flow within microchannels. This lays the foundation for future intelligent integrated diagnostic and therapeutic platforms (53, 54).

### 3.4 Operational modes and therapeutic strategies

Depending on clinical requirements and technological maturity, intra-articular PBM may follow distinct operational models, with current applications primarily focusing on short-term interventional and long-term implantable approaches. Short-term intervention employs ultra-fine flexible optical fibres (typically several hundred micrometres in diameter) as “optical conduits”. These are introduced into the joint cavity via minimally invasive techniques such as acupuncture needle or arthroscopic puncture, delivering single or limited-session targeted irradiation to cartilage defect areas and proliferative synovium. This strategy combines the biological effects of PBM with medical devices, aiming to promote post-operative repair, reduce inflammatory responses, and inhibit KOA progression (12). This approach presents a relatively low technical threshold and facilitates clinical translation. Long-term implantation involves surgically placing an integrated micro-illumination system (containing LEDs, power supply, and control unit) into the joint cavity via minimally invasive surgery, enabling repetitive or continuous low-dose illumination over weeks or even months. This “dynamic disease management” model enables continuous regulation of the intra-articular environment, particularly delivering timely intervention during acute inflammatory flare-ups, with the potential to fundamentally alter the course of KOA (55). With the advent of the intelligent era, future developments may evolve towards integrated diagnostic and therapeutic platforms. “Closed-loop” systems incorporating biosensing technology represent the cutting edge of this field (56). Within this framework, the intra-articular light source not only emits photonic energy but also integrates micro-sensors for detecting inflammatory markers, pH levels, or mechanical stress (57). Upon sensing biological signals indicative of acute KOA exacerbation, the system automatically initiates phototherapy. For instance, elevated TNF-α levels could trigger an anti-inflammatory phototherapy protocol, thereby achieving genuinely personalised, on-demand treatment.

## 4 Discussion

### 4.1 Potential advantages of intra-articular PBM for KOA

The proposal of intra-articular PBM represents not merely a circumvention of the limitations of percutaneous PBM techniques, but an innovation in therapeutic philosophy. By positioning the light source within the joint cavity, intra-articular PBM offers a distinct set of advantages and presents new opportunities to advance KOA management from “symptom relief” towards “functional restoration”.

#### 4.1.1 Overcoming physical barriers to achieve efficient treatment of deep tissue

The primary limitation of percutaneous PBM lies in the exponential attenuation of light within tissue. Research indicates that near-infrared light may lose over 90% of its energy density after traversing just a few millimetres of tissue (10). This implies that achieving effective therapeutic doses in deep cartilage may require energy levels far exceeding safe thresholds for the epidermis, or result in treatment failure (11). The intra-articular PBM strategy circumvents this physical barrier entirely. By directly introducing optical fibres into the joint cavity via acupuncture delivery techniques, light energy can be precisely delivered with minimal loss to target cartilage surfaces, synovium, or menisci. This not only ensures the affected area receives optimal energy density within the biologically stimulating window to maximise therapeutic efficacy, but also avoids the risk of thermal injury to the epidermis and subcutaneous tissues associated with high-energy transdermal irradiation (12, 58).

#### 4.1.2 Precision targeted therapy

The pathological changes in osteoarthritis are not uniformly distributed within the joint. Intra-articular PBM affords a degree of spatial selectivity. Under musculoskeletal ultrasound guidance, clinicians can precisely focus the light spot on specific areas of cartilage defect, inflamed and proliferating synovium, or damaged meniscus, without disturbing surrounding healthy tissue (12). This “point-to-point” precision intervention aligns closely with modern medicine’s principles of personalised, targeted therapy. For instance, it enables anabolic irradiation of degenerative cartilage margins to promote repair, while simultaneously administering anti-inflammatory irradiation to localised synovial inflammation. This achieves synergistic “anti-inflammatory and repair-promoting” effects – a level of targeting unattainable with diffuse transcutaneous PBM.

#### 4.1.3 A synergistic therapeutic platform transcending single PBM

The most compelling potential of the intra-articular PBM system lies in its capacity as a multifunctional platform, capable of deep integration with multiple cutting-edge therapies to generate synergistic effects where “one plus one is greater than two”. The intra-articular PBM system can serve as a trigger for light-controlled drug release. For instance, by encapsulating drugs (such as anti-inflammatory agents or growth factors) within light-sensitive nanocarriers and pre-injecting them into the joint cavity. Upon exposure to specific wavelengths of light, structural changes in the nanocarriers trigger precise drug release at specific times and locations (59). This temporally and spatially controlled “on-demand delivery” model significantly enhances drug utilisation while minimising systemic side effects. Mitochondrial reactive oxygen species (mROS) play a pivotal role in KOA progression. Research has developed a mitochondrial-targeted, NIR-responsive Mn_3_O_4_@PDA@Pd-SS31 nanozyme, which efficiently scavenges mROS, restores impaired mitochondrial function, and promotes cartilage regeneration, offering a promising therapeutic approach for KOA (60). Although gene therapy applications for KOA face challenges such as low delivery efficiency and high off-target risks, intra-articular PBM can enhance gene transfection via photothermal effects. For instance, co-injecting gene vectors with photothermal conversion materials (such as gold nanorods) into the joint followed by near-infrared light irradiation. The photothermal effect transiently increases cell membrane permeability, thereby significantly enhancing the uptake efficiency of therapeutic genes by target cells (61). This approach paves the way for safe and efficient intra-articular gene therapy.

### 4.2 Challenges in intra-articular PBM for KOA

Although intra-articular PBM offers an exciting new paradigm for OA management, numerous challenges remain on its path from laboratory to widespread clinical application. Objectively analysing these limitations and proposing feasible solutions is crucial for the healthy development of this field.

#### 4.2.1 Technical and engineering challenges

Achieving a stable, reliable, and long-term intra-articular PBM system presents a complex biomedical engineering challenge. An ideal implantable device must be compact to avoid impairing joint mobility while highly integrating light sources, power supplies, control units, and even sensors. Current technical hurdles include further miniaturising LED chips while maintaining sufficient output power and ensuring the durability of flexible circuits under repeated bending (52). The joint cavity presents a dynamic environment filled with synovial fluid and subject to continuous motion, demanding long-term mechanical and functional reliability from implanted devices. Energy supply constitutes a core bottleneck for long-term implantation strategies. Microbatteries possess limited energy density, struggling to sustain months or years of therapy. Whilst wireless energy transfer technologies (such as near-field communication) offer potential solutions, their transmission efficiency, penetration depth, and thermal effects on surrounding tissues require further optimisation (56). Developing energy harvesting systems, such as those utilising kinetic energy generated by joint motion, represents a highly promising future direction. Devices must match the mechanical properties of soft joint tissues, such as cartilage and synovium. Excessively rigid implants may cause cartilage wear, soft tissue irritation, or a foreign body sensation (62). Consequently, developing soft, stretchable encapsulation materials and device structures with tissue-like mechanical characteristics is key to achieving biomechanical compatibility.

#### 4.2.2 Biological and safety considerations

The biological safety of implanting electronic devices within joint cavities for sustained energy delivery must undergo rigorous evaluation. Although PBM is considered a low-risk therapy, prolonged and repeated intra-cavitary irradiation warrants vigilance regarding potential side effects, such as cumulative effects of low-grade thermal injury or unintended biological modulation of normal chondrocytes and synovial cells (63). Determining the therapeutic window—the energy density range yielding efficacy without causing harm—is paramount. Any implant triggers an organismal foreign body reaction, potentially leading to fibrous capsule formation. This encapsulation isolates the device, impairs light transmission and therapeutic efficacy, and may exacerbate joint fibrosis (64, 65). Optimising device surfaces with biocompatible coatings to minimise immune recognition and rejection responses represents a key strategy for enhancing biocompatibility. For intra-articular PBM, consensus standards remain elusive regarding optimal wavelength, power density, energy density, and treatment frequency (10). These parameters are likely to vary across different stages of KOA, pathological phenotypes (inflammatory or degenerative), and individual differences. Future research requires establishing parameter databases through large-scale, rigorous preclinical and clinical studies.

#### 4.2.3 Clinical translation barriers

The process of developing a market-ready medical product from a laboratory prototype is lengthy and fraught with challenges. Intra-articular PBM systems are usually combination products that integrate devices with pharmaceuticals or biomolecules. This entails navigating more complex and stringent approval processes with regulatory bodies such as the Medical Products Administration, requiring extensive data on safety, efficacy, and quality control (66). The development of miniaturised, implantable medical devices and the establishment of production lines compliant with Good Manufacturing Practice (GMP) standards incur substantial costs. This may result in prohibitively high final product prices, impacting accessibility, whilst cost reduction necessitates breakthroughs in manufacturing processes and economies of scale. Furthermore, promoting an entirely novel therapeutic paradigm requires altering established clinical practices, with clinicians requiring specialised training to master implantation and operational techniques. Concurrently, explaining the device’s principles, benefits, and risks to patients and obtaining their informed consent constitutes an indispensable aspect of clinical rollout.

### 4.3 Future research directions

To advance the clinical translation of intra-articular PBM, future research should address several pressing priorities. First, a standardized system of treatment parameters must be established by systematically identifying the optimal wavelength, energy density, and treatment frequency for different KOA phenotypes. Second, efforts should concentrate on developing next-generation devices with high biocompatibility and long-term stability, overcoming engineering challenges such as flexible and degradable light-emitting materials, reliable encapsulation technologies, and long-lasting power solutions. At the same time, rigorous clinical trials must be designed and conducted to verify the therapy’s efficacy and safety, providing high-level evidence for subsequent clinical application. Building on this foundation, the gradual exploration of intelligent integration with biosensing technologies and controlled drug delivery systems should be pursued to lay the groundwork for personalized, adaptive, closed-loop therapy (57, 67). Following this progressive, staged research and development pathway—from parameter optimization and hardware innovation to clinical validation and system integration—will allow intra-articular PBM to evolve from a laboratory concept into a mature therapy capable of meaningfully transforming the clinical management of KOA.

## 5 Conclusion

This review highlights intra-articular PBM as a transformative, minimally invasive strategy for KOA, shifting from palliative care to targeted, disease-modifying intervention. By delivering light directly into the joint via platforms such as OFA, this approach overcomes the tissue penetration limits of transcutaneous PBM, enabling precise modulation of the articular microenvironment. Mechanistically, PBM activates CCO, triggering synergistic anti-inflammatory, immunomodulatory (e.g., macrophage polarization), antioxidant, and pro-autophagic responses that collectively protect cartilage and subchondral bone. Beyond standalone therapy, intra-articular PBM serves as a versatile platform for integration with light-triggered drug delivery and regenerative strategies. While translational challenges in device durability and biocompatibility remain, interdisciplinary advances are paving the way for its clinical implementation, offering a promising pathway toward achieving long-term disease modification in KOA.
